# Stability and Lability of Parental Methylation Imprints in Development and Disease

**DOI:** 10.3390/genes10120999

**Published:** 2019-12-02

**Authors:** Sabina Farhadova, Melisa Gomez-Velazquez, Robert Feil

**Affiliations:** Institute of Molecular Genetics (IGMM), CNRS, University of Montpellier, 1919 route de Mende, 34293 Montpellier, France; sabina.farhadova@igmm.cnrs.fr (S.F.); mgomez@igmm.cnrs.fr (M.G.-V.)

**Keywords:** DNA methylation, histone methylation, *Polycomb*, genomic imprinting, ICR, DMR, imprinting disorders, evolution

## Abstract

DNA methylation plays essential roles in mammals. Of particular interest are parental methylation marks that originate from the oocyte or the sperm, and bring about mono-allelic gene expression at defined chromosomal regions. The remarkable somatic stability of these parental imprints in the pre-implantation embryo—where they resist global waves of DNA demethylation—is not fully understood despite the importance of this phenomenon. After implantation, some methylation imprints persist in the placenta only, a tissue in which many genes are imprinted. Again here, the underlying epigenetic mechanisms are not clear. Mouse studies have pinpointed the involvement of transcription factors, covalent histone modifications, and histone variants. These and other features linked to the stability of methylation imprints are instructive as concerns their conservation in humans, in which different congenital disorders are caused by perturbed parental imprints. Here, we discuss DNA and histone methylation imprints, and why unravelling maintenance mechanisms is important for understanding imprinting disorders in humans.

## 1. Introduction

Epigenetic modification of the genome by cytosine methylation—which in mammals is found at CpG (cytosine-guanine) dinucleotides—plays diverse roles in the control of gene expression [1,2]. It is important for the repression of developmental genes in the embryo, particularly of genes expressed in the germline only [3,4]. CpG methylation contributes also to the somatic repression of endogenous retroviruses (ERVs) [5]. In female embryos, in addition, cytosine methylation is essential in the allelic repression of X-linked genes. This occurs through the epigenetic process of X chromosome inactivation (XCI), which randomly inactivates one of the two X chromosomes in female embryos [6]. Perturbations in the establishment, or maintenance, of DNA methylation patterns cause different pathologies, and methylation changes contribute to cancer as well [7].

Our emphasis is on a different kind of allelic gene expression controlled by DNA methylation, namely genomic imprinting, and how perturbation of this epigenetic phenomenon leads to complex diseases in humans [8,9,10]. Genomic imprinting mediates a deterministic type of mono-allelic expression, dictated strictly by the parental origin of the gene [11]. Some imprinted genes are always expressed from the maternally-inherited allele, and others, always from the paternal copy. Extensive studies in mice and humans have identified about 200 imprinted protein-coding genes, and a growing number of non-coding RNAs (ncRNAs) are found to be controlled by genomic imprinting as well [9]. Other eutherians have been studied less extensively, but are thought to have similar numbers of imprinted genes [12].

Many imprinted genes display a mono-allelic pattern of expression that is tissue-specific. This indicates that their allelic dosage of expression is likely important in specific lineages, particularly in the placenta and in brain, in which most genes show imprinted expression [13]. Genetic studies in mice have proven that the dosage control conferred by imprinting is indeed essential for diverse biological processes, including fetal growth, placental development and function, endocrine and metabolic functions, and brain development and behavior [13,14]. Recent reviews nicely present the biological roles of imprinted gene expression, and discuss the reasons for which this epigenetic phenomenon evolved in eutherians, about 170 million years ago [12,13,14].

A common feature of imprinted genes on autosomal chromosomes is that they are organized in conserved chromosomal domains that comprise multiple genes. Essential for the acquisition of mono-allelic expression at these large domains (up to several megabases, Mb) during embryonic development are the ‘imprinting control regions’ (ICRs) [15]. The allelic gene expression at each imprinted gene domain is controlled by one such ICR. Briefly, ICRs are key regulatory elements whose sequences are G + C rich, and most correspond to CpG islands [16]. All ICRs are marked on one of their two parental alleles by germline-acquired DNA methylation. These parental methylation ‘imprints’ originate from either the maternal germline (at the so-called ‘maternal ICRs’) or the paternal germline (at ‘paternal ICRs’) [17] and cover several kilobases of DNA [18].

In male germ cells, DNA methylation imprints become established during fetal development, several days after the demethylation of the genome in primordial germ cells (PGCs). At the time of birth, there is full DNA methylation at paternal ICRs in all the germ cells, which persists throughout the male’s lifetime [19,20]. Methylation imprints at maternal ICRs are acquired in the adult animal, during the final stages of oogenesis when oocytes expand in size [21]. Despite this divergence in timing [22], both in male and female germ cells the acquisition process involves de novo DNA methyltransferase (DNMT)3A [23,24]. DNMT3L, a DNMT-like protein, is required for imprint acquisition as well, particularly in the female germline [25]. This catalytically-inactive DNMT plays an important role in ‘selecting’ the chromosomal regions to become methylated. In mouse oocytes, DNMT3L guides CpG methylation towards chromatin regions that are transcriptionally active and are enriched in transcription-linked H3 lysine-36 tri-methylation (H3K36me3), but are devoid of H3-lysine-4 methylation [25,26,27,28,29]. Recent texts discuss the establishment of DNA methylation imprints in early male germ cells and oocytes [17,30], and this is not the emphasis of the current review.

After fertilization, germline-acquired DNA methylation imprints are stably maintained at most imprinted loci, such that the methylated allele is stable kept methylated and the non-methylated allele does not acquire de novo DNA methylation. This somatic maintenance process is a hallmark of genomic imprinting [17]. It is essential to bring about the parental allele-specific expression of close-by genes later on in development, often in a tissue-specific manner, and through mechanisms that differ between imprinted gene domains [15].

Thus, the specificity of imprinting depends not only on methylation acquisition in the germline, but also on their exceptional maintenance during embryonic development [17,31]. In fact, more than a thousand CpG islands become methylated during oogenesis, but after fertilization, only some twenty of these maintain their maternal methylation imprint during preimplantation development [20,21]. These are the maternal ICRs. In order to understand this specificity, it is important to understand the embryonic maintenance of differential methylation at ICRs [17], a process that strictly requires the maintenance methyltransferase DNMT1 [32]. Many other factors contribute to the process as well [33], at all, or subsets of, ICRs (Table 1), which we will discuss below.

The emphasis of our review is on DNA methylation and histone methylation imprints, their somatic maintenance during development, and on how their epigenetic perturbation affects gene expression and phenotype. We will describe the often-constitutive, but sometimes lineage-specific, maintenance of methylation imprints. Linked to this theme, is the question of how pathological epigenetic alterations come about in human disease [9,33,54]. During the last years, the rapidly expanding body of knowledge has helped to understand the embryonic stability of methylation imprints, and why this process may become perturbed in pathological conditions.

## 2. Embryonic Stability of Germline-Acquired DNA-Methylation Imprints 

**Figure 1:**
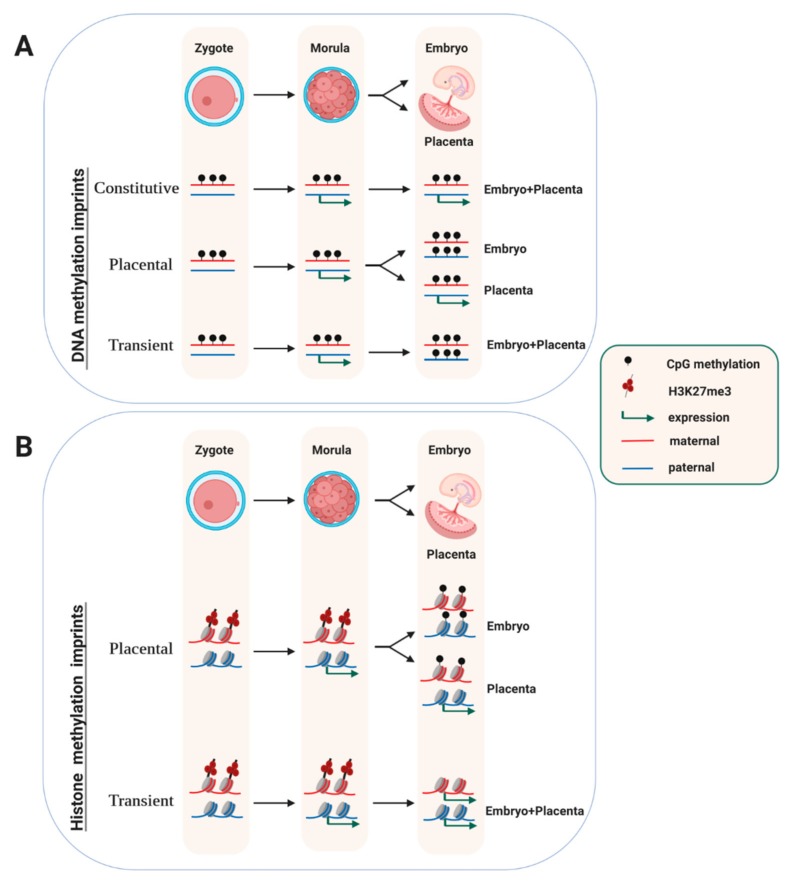
Somatic maintenance of oocyte-derived DNA-methylation and H3 lysine-27 tri-methylation (H3K27me3) imprints. (**A**) At most maternal ‘imprinting control regions (ICRs)’, the differential DNA methylation state is maintained in all somatic lineages (‘Constitutive’). At some, however, it persists during extra-embryonic development only and is lost in the embryo proper (‘Placental’). At a few differentially methylated regions (DMRs), the differential methylation and expression status is short-lived and lost in both the embryo and the placenta (‘Transient’). (**B**) Oocyte-derived H3K27me3 imprints give rise to a paternal allele-biased gene expression that is labile and is lost before implantation at most loci (‘Transient’). At only a handful of genes, the allelic expression persists in the trophoblast (‘Placental’), through somatic acquisition of repressive DNA methylation on the maternal allele. Figure 1 Figure 2 Figure 3 were created using Biorender.com.

At most imprinted domains in the mouse (i.e., 22 out of 25 well-studied domains), the imprint is acquired during oogenesis [17]. At only three imprinted domains, the germline imprint is acquired during spermatogenesis. Following fertilization, the parental methylation marks at ICRs are maintained throughout pre-implantation development, and often throughout post-implantation development as well. This classical pattern of constitutive imprint maintenance at germline differentially methylated regions (gDMRs) (Figure 1A) contrasts with the dramatic, genome-wide methylation changes that occur during the first few days of development [2]. Following fertilization, both the parental genomes rapidly loose most of their DNA methylation on the genome. This active process involves a ten eleven translocation (TET) protein, TET3, which catalyzes the oxidation of 5-methylcytosine into 5-hydroxymethylcytosine, which is followed by additional chemical transitions and less understood DNA repair mechanisms, eventually leading to a non-methylated cytosine [55]. Additionally, there is a passive, replication-linked, loss of DNA methylation, which explains the global methylation reduction particularly on the maternal genome [56]. Interestingly, the extent of DNA demethylation in the post-fertilization embryo differs between species. The global methylation losses are much more pronounced in mice than in humans [57].

Differentially methylated regions (DMRs) with germline-acquired methylation are fully protected against the genome-wide waves of active and passive DNA demethylation during the first few cell cycles. Later in development, during gastrulation, there is acquisition of extensive de novo DNA methylation along the most of genome, mediated by the de novo DNA methyltransferase DNMT3A/B [1]. Again here, imprinted loci are protected, with the non-methylated alleles of ICRs being kept non-methylated [17,58].

Genome-wide studies have shown that not all gDMRs are maintained in the embryo [11]. In one genetic screen that used the dependence of methylation acquisition in oocytes on DNMT3L as a criterion [59], several loci were identified at which the allelic methylation status was lost in adult tissues, through acquisition of biallelic DNA methylation (Figure 1A). The differential methylation at these germline DMRs (gDMRs)—though transient—can nevertheless be functionally important. At the mouse *Gpr1* gene, in the early embryo, a maternal gDMR mediates transient expression of a long non-coding RNA (called ‘Liz’) from the paternal chromosome only. This allelic lncRNA, in turn, brings about paternal DNA methylation at a close-by protein-coding gene (i.e., *Zdbf2*), and this confers imprinted gene expression later in development [60,61]. Whereas in the embryo proper, the *Gpr1* gDMR becomes methylated on both the parental chromosomes, in the placenta its allelic methylation state persists. Thus, at this locus, the maintenance process is different in the trophoblast as compared to the embryo proper. A similar transient maintenance of maternal methylation occurs at several other imprinted loci as well [59,62], including at *Fkbp6*, a gene important in germ cell development [63]. 

Trophoblast-specific imprinted expression is particularly prevalent in humans. At hundreds of loci, oocyte-derived imprints are maintained until the blastocyst stage, and after implantation, persist in the placenta only [62,64,65,66,67]. Though still poorly understood, and not always associated with differential gene expression, this maintenance pattern agrees with the known role of imprinting in placental functions [62,68]. There is not much overlap, however, with placental-specific imprinted expression in mice. The phenomenon could therefore undergo rapid evolution, with divergent roles in primates and rodents [13,68]. The prevalent placental-specific imprinting could be mechanistically linked to the less pronounced global DNA demethylation in the human preimplantation embryo, which would facilitate maintenance of a higher number of gDMRs during this critical developmental period [57].

Mono-allelic to biallelic DNA methylation switches occur in adult tissues as well. This concept first emerged from studies on the *Dlk1-Dio3* domain on mouse chromosome 12 [69]. The domain’s *Dlk1* gene encodes an antagonist of Notch signaling that plays diverse roles in development and is expressed from the paternal chromosome only in fetal neural cells [70]. Postnatally, *Dlk1* becomes expressed from both parental alleles, in neural stem cells and in niche astrocytes, and this is mediated by a mono-to-biallelic methylation switch at the domain’s ICR [71]. 

Differential DNA methylation states can be newly acquired during embryonic development as well. This occurs at the so-called ‘somatic DMRs’ (sDMRs), secondary DMRs that arise through the allelic functions of ICRs. sDMRs are formed through de novo DNA methylation, often guided by allelic lncRNAs that are expressed by the ICR [15]. Similarly as at the germline DMRs (ICRs), the allelic methylation is maintained throughout subsequent development at most sDMRs [15]. Therefore, mechanisms involved in the somatic maintenance of sDMRs are thought to be similar to those acting on gDMRs, a theme we will develop further below.

## 3. Transcription Factors, Histone Modifications, and Variant Histones in the Maintenance of Methylation Imprints.

ICRs show little DNA sequence homology between each other. Nevertheless, common factors are thought to ensure their imprint maintenance [17,33], see Table 1. Intriguingly, several ICRs are enriched in, or flanked by, tandemly repeated DNA sequences [72]. At the ICR of the *Dlk1-Dio3* domain, tandem repeats were found to contribute to the maintenance of its paternally-inherited DNA methylation [73]. Deletion of tandem repeats within, or close to, several other ICRs, however, did not impact methylation imprints [74,75].

DNMT1 is absolutely essential in DNA methylation maintenance, and maternal plus zygotic knockout leads to a complete demethylation of all ICRs in the zygote [32]. UHRF1 (also called NP95), a protein that binds hemi-methylated DNA and recruits DNMT1 following replication of the DNA, is essential for methylation imprint maintenance as well [35].

The expression in the oocyte of DPPA3 (‘developmental pluripotency-associated-3’, also called PGC7 and Stella) is critical for the early post-fertilization maintenance of imprinted DNA methylation, not only on the maternal, but also on the paternal genome. *Dppa3*−/+ maternal-null embryos showed methylation losses at several maternal (*Peg1*, *Peg3*, and *Peg10*) and several paternal ICRs (including *H19*) [44,45]. This SAP-like domain protein is suggested to prevent DNA demethylation through its binding to the C-terminal domains of TET2 and TET3, which would prevents these proteins’ DNA demethylation activity [76]. DPPA3 is specifically recruited to chromatin that is enriched in H3-lysine-9 dimethylation (H3K9me2), a repressive histone modification. This chromatin binding pattern has been proposed to explain the locus-specificity of its protective action against DNA demethylation [45].

In mammals, H3K9me2 is controlled by the lysine methyltransferase (KMT) G9A (also called EHMT2), together with its partner protein ‘G9A-like protein’ (GLP). G9A is highly expressed during oogenesis and in the post-fertilization embryo [77]. Oocyte-specific knockout of *G9a* resulted, as expected, in loss of H3K9me2 [77]. Unexpectedly, in the resulting oocytes and early embryos, there was no evidence for altered DNA methylation at ICRs, or genome-wide. This finding contradicts the idea that the protective effect of DPPA3 is mediated through recognition of H3K9me2 in the early embryo [77]. It complements recent studies on *G9a*−/− knockout embryos [78] in which the absence G9A KMT gave only a partial loss of methylation loss at one ICR (at the *Slc38a4* locus), and did not induce genome-wide losses in DNA methylation either [4]. Thus, G9A is not involved in the maintenance of DNA methylation imprints.

**Figure 2:**
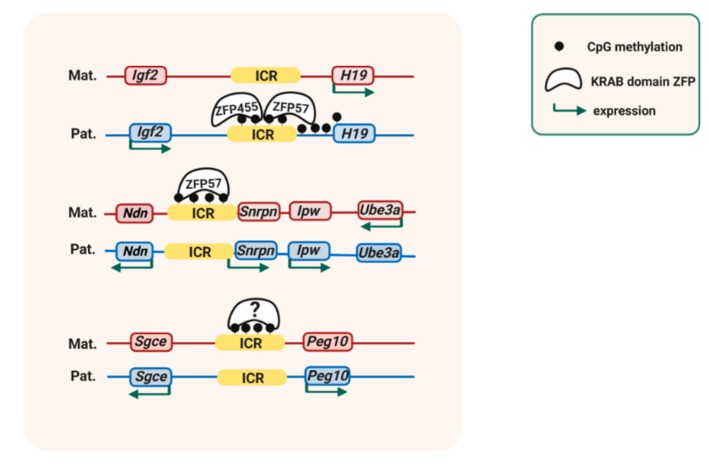
Methylation-imprint maintenance requires KRüppel Associated Box (KRAB)-domain zinc finger proteins. In the mouse, different imprinting control regions (ICRs) are bound by different KRAB domain zinc finger proteins (ZFPs) on the DNA-methylated allele, which maintains allelic DNA methylation and allelic gene expression. The ICR of the mouse *Igf2-H19* domain comprises binding sites for both ZFP57 and ZFP445, whereas the ICR of the *Snrpn* domain (‘Prader-Willi Syndrome domain’ in humans) has a recognition sequence motif for ZFP57 only. It is unknown which ZFP is recruited to the methylated allele of the ICR of the mouse *Peg10/Sgce* imprinted domain.

A strong contribution is provided by KRüppel-Associated Box (KRAB)-domain zinc finger proteins (ZFPs) that bind to methylated DNA sequence motifs [79] (Figure 2). The first-identified was ZFP57. Targeting of *Zfp57* in both the oocyte and the zygote led to loss of DNA methylation at many ICRs [37]. The imprinted *Igf2-H19* and *Igf2-receptor (Igf2r)* loci were unaffected, however, and stably maintained their allelic ICR methylation in the absence of ZFP57. Recessive *ZFP57* mutations affect a small sub-set of ICRs only in humans [38], which suggested that another KRAB-domain ZFP could control other ICRs in this species [80]. 

Genome-wide chromatin immunoprecipitation (ChIP) and RNA-seq studies in embryonic stem (ES) cells have explored the critical importance of ZFP57 in allelic gene expression [81]. Both in humans and mice, ZFP57 recognizes a 6-base-pair motif (TGCCGC) that is present at many ICRs [82]. In a recent studies on human ES cells and mouse embryos, Takahashi and colleagues [39] found that the KRAB-domain protein ZNF445 is a regulator of ICR methylation as well. Similarly as ZFP57, ZFP445 binds to the methylated alleles of a sub-set of the ICRs that include the ICRs of the *Igf2-H19* and *Igf2r* domains. It plays a much more important role in humans than in mice, in which it is recruited to only some ICRs, whereas in humans its sequence recognition motif is found at many ICRs [39]. 

**Figure 3:**
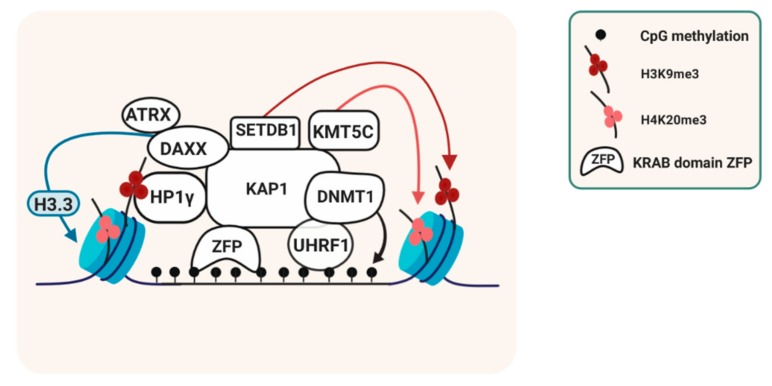
Protein complexes linked to the somatic maintenance of methylation imprints. The specific binding of KRüppel Associated Box (KRAB) domain zinc finger proteins (ZFPs) to the DNA-methylated alleles of imprinting control regions (ICRs) leads to KRAB-associated protein-1 (KAP1) recruitment, which facilitates the recruitment of chromatin remodeling and histone methylation proteins and of proteins involved in the maintenance of DNA methylation. H3 lysine-9 tri-methylation (H3K9me3) and H4K20me3 are both consistently associated with the DNA methylation imprint at ICRs, and the representation assumes that they are present within the same nucleosomes. There is also recruitment to the chromatin linked to the methylation imprint of variant histone H3.3, through the Alpha Thalassemia/Mental Retardation Syndrome X-Linked- Death Domain Associated Protein ATRX-DAXX chaperone complex.

ZFP57 and/or ZFP445 binding to ICRs controls methylation maintenance in different ways [80]. These proteins recruits the platform protein KAP1 (‘KRAB-associated protein-1’, also called TRIM28) [63]. This, in turn, leads to local recruitment of SETDB1 (ESET), a KMT that catalyzes H3K9 tri-methylation (H3K9me3) [39,42,63]. KAP1 also recruits the chromatin-structural protein HP1γ to the methylated alleles of ICRs [42]. In mouse cells, KAP1-deficiency leads to variable DNA methylation losses at different ICRs [46]. Loss of expression or reduced expression of SETDB1, similarly, leads to partial losses of DNA methylation at several ICRs as well [42,43]. The relative importance of HP1γ in imprint maintenance remains to be explored (Figure 3). 

An additional mode through which KRAB-domain ZFPs and KAP1 prevent loss of DNA methylation is through their interaction with DNMT1 and UHRF1, which is important for DNA methylation maintenance as well [32,35]. 

Yet-other proteins contribute to the somatic maintenance, albeit to a lesser extent (Table 1). These include the methyl-CpG-binding domain (MBD) protein MBD3, and MTA2, both components of the ‘nuclear remodeling and deacetylation’ (NuRD) complex. Their depletion in mouse cells gave partial methylation losses at the ICRs of the *Igf2-H19* and *Peg3* imprinted domains [36,51]. At the ICR of the human *IGF2-H19* domain, the methylated-CpG binding protein KAISO (also called Zinc Finger and Broad-cComplex, Tramtrack and Bric- à brac (BTB) Domain Containing-33, ZBTB33) contributes to the somatic maintenance of DNA methylation as well [40].

Other proteins are recruited to ICRs through specific chromatin modifications that are enriched on the allele that carries the DNA methylation imprint. There is allelic recruitment of the protein arginine methyltransferases PRMT5, which brings about symmetrical H4-arginine-3 dimethylation (H4R3me2s) specifically on the methylated alleles of ICRs. H4R3me2s, however, seems not involved in the somatic maintenance of DNA methylation [42]. Consistently, there is H4 lysine-20 tri-methylation (H4K20me3) enrichment on the DNA-methylated alleles as well [42,83]. This chromatin modification influences the timing of DNA replication [84], but whether this is the case at ICRs is unclear.

Chromatin associated with the DNA-methylated alleles of ICRs is enriched in histone H3 variant H3.3 [47]. H3.3 incorporation occurs replication-independently and requires the histone chaperone complex ATRX/DAXX. ATRX is specifically recruited to the methylated alleles of ICRs, possibly through association with the KAP1-recruited HP1γ. ATRX depletion in murine ES cells leads to loss of H3.3, loss of H3K9me3 and biallelic expression, at some imprinted loci only [47,85].

Recent mouse studies link N-terminal acetylation of proteins to the somatic maintenance of DNA methylation imprints [48]. N-α-acetyltransferase-10 (NAA10P, also called ARD1) is a nuclear acetyltransferase that plays diverse biological roles, including in cellular proliferation [86,87]. In humans, missense mutations had been linked to a severe growth-retardation syndrome, Ogden syndrome (OMIM 300855), in which babies present malformations and die at an early age [88]. *Naa10p*−/− mice show a partially penetrant embryonic lethality, due to a lack of the maternally transmitted NAA10P protein, and this gives highly abnormal placental development. The few surviving newborns had severe growth retardation and diverse brain defects [48]. Given the pleiotropic nature of these phenotypes, and the importance of maternal NAA10p expression for placentation [89], the authors suspected that imprinted genes could be perturbed. Indeed, in NAA10P-deficient embryos (and *Naa10p*−/− ES cells) several imprinted loci showed loss of ICR methylation and ‘loss of imprinting’ (i.e., biallelic expression) [48]. It is unclear how precisely NAA10P expression contributes to the somatic maintenance of imprints. However, this nuclear protein associates preferentially with the methylated ICR alleles [48]. It binds DNMT1, but does not acetylate this DNA methyltransferase [86]. These mechanisms, together with action of the UHRF1 [35], likely contribute to the efficient recruitment of DNMT1 to ICRs following replication [48]. It is not yet known whether methylation imprints are perturbed in Ogden syndrome as well.

ICRs are not the only sequences at which repressive DNA methylation and chromatin have to be stably maintained. A similar requirement concerns endogenous retroviruses (ERVs)—also called long-terminal-repeat (LTR) retrotransposons—that are scattered in thousands of copies across the genome. These ‘foreign’ DNA elements have to be kept repressed most of the time. Biochemical studies show that similar mechanisms maintain repressed chromatin and DNA methylation at ICRs and ERVs—particularly at intracisternal A particles (IAPs)—with involvement of the same nuclear complexes including specific KRAB domain ZFPs, KAP1, SETDB1, HP1γ, ATRX/DAXX, and variant histone H3.3 [33]. This new insight [33] may be relevant for how imprinting evolved, a process that occurred concomitantly with the acquisition of defense mechanisms against retrotransposons [33,90,91]. 

Individual components of the machinery can have different effects on ERVs and ICRs. An interesting example is ZFP57. In mouse stem cells, ZFP57 binds not only to the methylated alleles of many ICRs, but also to non-imprinted unique regions, and to LTR-containing transposable elements. In *Zfp57−/−* ES cells, KAP1 binding and H3K9me3 is completely lost at ICRs. At non-imprinted target regions, including ERVs, however, these chromatin features are unaffected. This suggests that here, ZFP57 acts redundantly together with other factors, including possible other KDZFPs [81,92].

## 4. Protection of ICRs Against *de Novo* DNA Methylation

For imprinted gene regulation it is essential to protect the non-methylated alleles of ICRs as well. Given that some gDMRs acquire de novo DNA methylation on the opposite allele in the developing embryo [59], an active process is needed to protect the non-methylated allele against de novo methylation at other ICRs [15]. All maternal ICRs comprise promoters that are either active, or transcriptionally poised, which explains why they are consistently marked by histone H3 lysine-4 trimethylation (H3K4me3) in embryonic stem and differentiated cells [93,94,95]. Mechanistically, this ‘active’ chromatin mark is essential in the protection against DNA methylation, because it prevents the DNMT3A-DNMT3L protein complex to access the chromatin-associated DNA [26,27]. In embryonic cells and tissues that lack detectable promoter activity at specific ICRs, the ICRs’ allelic H3K4me3 occurs in the context of so-called ‘bivalent chromatin’ [96], with co-presence of repressive H3 lysine-27 tri-methylation (H3K27me3) [93]. This consistent finding suggests a possible role of bivalent chromatin in protecting the unmethylated ICR alleles against de novo DNA methylation, not only in early embryonic cells, in which the bivalent chromatin state was described first [94,96], but also in differentiated cells [93].

Protection against methylation is particularly critical before implantation and the formation of the embryonic lineages. Not surprisingly, therefore, several studies have shown involvement of pluripotency-associated transcription factors (TFs) in imprint maintenance. The zinc finger protein ZFP42 (also called REX1) is a stem cell marker highly expressed in the preimplantation embryo. *Zfp42*−/− blastocysts showed gains of methylation at the ICRs of the *Peg3* and *Gnas* imprinted domains, on the normally unmethylated alleles. Concordantly, the protein preferentially binds to the unmethylated alleles of these two ICRs [97]. The pluripotency-associated TFs POU5F (also called OCT4) and SOX2 are both recruited to the unmethylated allele of the ICR of the *IGF2-H19* domain. In some patients with the IGF2-linked fetal overgrowth syndrome Beckwith–Wiedemann Syndrome (BWS, OMIM 130650), mutations in the POU5F/SOX2 binding sites are linked to gains of methylation at this paternal ICR [98,99]. At the *IGF2-H19* ICR, there is also recruitment to the unmethylated allele of CCCTC-binding factor (CTCF), a chromatin protein that mediates long-range structural interactions between different regulatory regions [100]. In the mouse, deletion of the four CTCF binding sites led to aberrant methylation at the ICR during early embryogenesis [101,102].

At the somatic DMR of the *Meg3* lncRNA gene at the *Dlk1-Dio3* imprinted domain, the KRAB domain zinc finger protein ZFP281 facilitates promoter activity on the maternal chromosome both in embryonic and trophoblast cells. It recruits MLL protein complexes to the chromatin, which brings about H3K4me3, and also interacts with AFF3, a component of the transcription elongation complex [103,104,105].

It remains unclear how precisely pluripotency-associated TFs protect against de novo DNA methylation. This could be by bringing about H3K4me3, which prevents recruitment of de novo DNMTs. TFs could also recruit TET proteins, and hence, induce continued DNA demethylation. Such a scenario has been proposed for the pluripotency-associated TF ‘PR domain containing regulator-14’ (PRDM14), which interacts with TET1 and TET2 and thereby regulates active DNA demethylation [106,107]. Its precise role in germline acquired DNA methylation is unclear, and has been complicated to explore because of the essential roles of PRDM14 in gametogenesis [108]. However, several-fold overexpression of PRMT14 in ES cells gave rise to partial methylation losses at multiple ICRs [106], underlining the likely importance of the dosage of this pluripotency-associated factor in imprint maintenance.

## 5. Transient Maternally-Inherited Imprints that are Independent of DNA Methylation 

Imprinting mechanisms independent of DNA methylation are thought to exist, in which chromatin modifications other than cytosine methylation are inherited from the germ cells and give rise to allelic expression in the early embryo [22]. For instance, it had been found that some imprinted gene loci did not display any differential DNA methylation in the embryo [57]. Transgenic experiments on the ICR of the murine imprinted *Igf2-H19* locus have given interesting insights as well [109]. This paternal ICR is fully methylated in sperm. When in transgenic experiments it was inserted into ectopic loci, however, it did not become methylated during spermatogenesis, but acquired paternal methylation in the preimplantation embryo only [109,110]. Somehow, another mark inherited from sperm was recognized in the early embryo to guide the de novo methylation machinery to the right parental allele. This intriguing effect depends on the ectopic locus’ context. When the ICR was inserted into the *IgH* locus of the mouse, acquired and maintained DNA methylation exactly as at the endogenous locus [111].

Female mice in which DNMT3L was no longer expressed during oogenesis generated offspring, in which some imprinted genes were nevertheless repressed on the maternal allele. In offspring of these *Dnmt3L*−/− females, maternal DNA methylation was variably re-established during embryogenesis at several ICRs [112]. This included the maternal ICR of the imprinted *Snrpn* locus [113]. Somehow, the methylation machinery recognized the right parental allele in some of the *Dnmt3L*−/+ embryos. A similar mechanism was suggested to act on the human locus, based on an apparent lack of methylation at the *SNRPN* ICR in mature oocytes [114]. 

These and other observations suggested the existence of germline acquired mark(s) other than DNA methylation, which indicate the parental origin of the allele and can be recognized post-fertilization [5,22]. Recent studies explored this intriguing idea in a genome-wide manner, by screening for gene loci that showed hypersensitivity to nucleases on the paternally-inherited allele at the zygote and morula stages and that had no differential DNA methylation [115]. The study then compared these regions with oocyte- and early-embryo genome-wide ChIP data sets [116] on H3 lysine-27 tri-methylation (H3K27me3), a repressive modification controlled by the Polycomb Repressive Complex 2 (PRC2). Many genes showed expression and nuclease hypersensitivity on the paternal allele mostly, and chromatin at these genes was marked by oocyte-derived repressive H3K27me3 on the maternal allele. At only some genes, the paternal bias in expression persisted till the blastocyst stage, but was lost subsequently. In female embryos, these included the lncRNA gene *Xist*, which is repressed on the maternally allele by the oocyte-derived H3K27me3 [117]. The functional importance of this maternal H3K27me3 was confirmed by overexpressing KDM6B, a demethylase that removes methyl groups from H3-lysine 27. This induced loss of H3K27me3 and biallelic gene expression at the loci analyzed [115]. Combined, these recent data evoke a transient mode of imprinted gene expression mediated by oocyte-derived H3K27me3 imprints (Figure 1B, ‘transient’). 

Recent studies show that at eight H3K27me3-marked protein-coding genes, the paternal expression persists in the placenta, while in the embryo proper these genes become fully repressed [115,118]. This placental-specific imprinted expression is no longer mediated by H3K27me3—which is lost in the extra-embryonic ectoderm [118]—but involves acquisition of de novo DNA methylation on the maternal allele. In the embryo itself, both parental alleles acquire DNA methylation. At this class of placental-specific genes (Figure 1B, ‘placental’), interestingly, the oocyte-derived H3K27me3 co-localizes with ERV-LTR insertions, whose allelic activity drives transcription across the paternal allele in the extra-embryonic lineage. This also somehow protects the paternal allele against DNA methylation (Figure 1). How this class of genes become DNA-methylated specifically on the maternal allele remains to be determined, but this seems independent of the original, oocyte-derived, H3K27me3 imprint [118].

The transient, H3K27me3-mediated imprinted gene expression is conserved in humans, at least in part [119]. The biological roles of the H3K27me3-linked imprinted expression, and whether it contributes to human disease, should therefore be interesting to explore. This in particular, for the small group of H3K27me3-marked genes that maintains their paternal allele-specific expression in the extra-embryonic tissues.

## 6. New Insights into Maintenance Mechanisms from Imprinting Disorders

Although the mouse has provided valuable insights, it is essential to also explore human cells and tissues. Genomic imprinting as a mechanism is conserved amongst eutherian species, but some genes are imprinted in humans only, and others only in mice [12]. There are general gene-regulatory differences between the two species as well [120]. Following fertilization, there is a less pronounced genome-wide loss of DNA methylation in humans than in mice [57]. The embryonic genome becomes transcriptionally activated at the eight-cell stage only in humans, compared to the two-cell stage in mice. The latter difference provides a longer time-frame in humans during which maternal factors can exert their effects, before the embryonic genome takes over. In the context of these species’ differences, it is interesting to note that many more genes are imprinted in the human placenta than in the mouse placenta [62], a difference that could be caused by differential imprint maintenance in the trophoblast as well.

Consequences of genetic mutations and of environmental cues can differ between species as well [9]. ZFP57 controls a high number of ICRs in the mouse [37,81], but in humans only a minority of the ICRs have a ZFP57 binding site [121]. A related KRAB domain protein, ZFP445, is much more important in humans [39]. The imprinting disorder ‘transient neonatal diabetes mellitus’ (TNDM, OMIM 601410) is caused by mutations in the human *ZFP57* gene [38] and presents a relatively minor clinical phenotype. In the mouse, ZFP57 controls the majority of ICRs, and *Zfp57*−/− knockout mice are embryonic lethal, with grossly perturbed imprinted expression [37,39].

Imprinting-related disorders currently include twelve congenital diseases characterized by perturbed development, growth, and metabolism [9]. Though each imprinting disorder is linked to one chromosomal domain mostly, these complex diseases show intriguing clinical overlaps. This has evoked the hypothesis that imprinted domains are involved in common biological processes, and may become perturbed in concert in human disease contexts. Recent data provide evidence for both these propositions [54]. For instance, several imprinted genes (*IGF2, INS, IGF2R,* and *GRB10*) encode proteins that are part of the insulin/insulin-like growth-factor (IGF) signaling pathway involved in growth and metabolism [13]. Imprinted genes are also co-regulated in specific cells and tissues [122]. Perturbation of specific imprinted genes was found to affect expression levels of many other imprinted- and non-imprinted loci as well. Some imprinted gene loci produce transcription factors, or chromatin regulatory proteins, that control the expression of other imprinted loci [123]. Additionally, imprinted lncRNAs were reported to influence imprinted gene expression in trans, at other imprinted domains [122,124,125,126]. These different observations have led to the concept of an ‘imprinted gene network that involves multiple co- and trans-regulatory links between genes [123,126].

Besides uniparental disomies (UPDs) and other genetic events, imprinting disorders can be caused by aberrant DNA methylation changes at ICRs or somatic DMRs [54]. In some patients, loss of DNA methylation is detected at multiple ICRs. These multi-locus disturbances’ are frequently observed in TNDM, in the overgrowth syndrome ‘Beckwith–Wiedemann Syndrome’ (BWS, OMIM 130650), and in the growth-restriction syndrome ‘Silver–Russell Syndrome’ (SRS, OMIM 180860) [9,54]. It will be a challenge to unravel why, in some cases, multiple ICRs lose their DNA methylation imprint in the same early embryo, to what extent this phenotype is caused by genetic predisposing factors, and whether stochastic mechanisms contribute to the highly variable nature of multi-locus disturbances as well. 

So far, only few causes of multi-locus imprinting disturbances have been pinpointed in patients. These include genetic mutations in *ZFP57,* whose perturbation leads to TNDM, through hypo-methylation and loss of imprinting at the *PLAGL1* transcription factor gene. In some TNDM patients, hypo-methylation occurs at one or more other ICRs as well [9,121]. 

A study on five selected families with multi-locus imprinting disturbance pinpointed genetic alterations in a gene called NLRP5, in the mothers of children that presented either SRS or BWS [50]. These maternal-effect NLRP5 variants correlated with methylation losses at a small number of ICRs in the affected children. The observed variable nature and mosaicism of the observed methylation losses explained why some of these children presented SRS, and others, BWS [50]. NLR family pyrin-domain proteins (NLRPs) are mostly-cytoplasmic proteins that are expressed in oocytes and in preimplantation embryos. Besides NLRP5, maternal variant mutations in two other members (NLRP2 and NLRP7) are associated with imprinted DNA methylation perturbation. It is unclear how NLRP5 contributes to imprint maintenance, but this could be linked to its recently reported association to specific transcription factors [54]. 

In a recent study whole-exome sequencing was performed on 38 different families with multi-locus imprinting defects. This confirmed the role of maternal effect gene NLRP5, identified novel maternal effect variants in NLRP2 and NLRP7, and led to the discovery of several other putative maternal-effect protein variants as well [127]. Amongst these, interestingly, is UHRF1, the co-factor that recruits DNMT1 to hemi-methylated DNA following replication. 

Several imprinted domains seem resistant to the multi-locus methylation losses observed in BWS, SRS, and TNDM [54]. Their more stable methylation maintenance at ICRs could be linked to additional mechanisms that evolved locus-specifically. One example is the *SNRPN* domain on human chromosome 15q11-13, at which genetic perturbations cause the neurodevelopmental syndrome Prader–Willi Syndrome (PWS, OMIM 176279). The retinoblastoma (Rb) binding proteins ARID4A (also called RBBP1) and ARID4B (also called RBBP1L1) are co-recruited to the DNA-methylated allele of the ICR of this domain [52]. Both these RB-like proteins have a DNA binding domain, and comprise TUDOR and chromo domains, which mediate binding to methylated H4 and H3 histones. Deletion of both the genes in the mouse leads to reduced H3K9me3 and H4K20me3 and loss of DNA methylation at this ICR [52]. 

Different chemical exposures and environmental conditions, such as in vitro embryo culture, can induce DNA methylation and expression changes at imprinted loci as well [33,128]. In experimental animal studies, often, the most pronounced effects are observed in the placenta, which suggests that the trophoblast is particularly susceptible to environmental effects [33,129].

In cohorts of babies conceived through assisted reproduction technologies (ART), there is a higher occurrence of imprinting disorders compared to naturally-conceived babies (reviewed in [54]). However, the absolute risk of babies presenting with an imprinting disorder remains extremely low. The relative increase in ID frequency could be linked to the compromised fertility of the couples that seek help from the clinic, or, alternatively, is caused by the reproduction procedure(s) themselves [129]. Novel approaches, including genome-wide (epi)genomic studies are required to disentangle the relative contributions of genetic factors, stochastic events, and environmental cues in the aberrant methylation changes in imprinting disorders, and as a consequence of environmental cues.

## 7. Conclusions

Through diverse genome-wide studies valuable new insights have been generated during the last years. Most notably, it has become apparent that imprinted gene expression is different in the trophoblast (placenta) compared to the embryo proper, with the discovery of many new gene loci that show imprinted expression in the placenta only, in humans often in a polymorphic manner. How differential methylation imprints can be maintained specifically in the placenta remains largely unknown, but one can assume that this involves factors that are expressed in the trophoblast only. So far, studies on maintenance mechanisms have focused on the embryo and on embryonic cells, and new maintenance factors have been pinpointed (Table 1). Given the growing importance of placental-specific imprinting, it should now be relevant to develop extra-embryonic cell-based models as well.

A disease-related breakthrough of the last years is that maternal ICRs often show loss of methylation in concert in different pathologies, suggesting that common factors could be perturbed. So far, however, few such factors are known in disease. By combining genome sequencing studies -which are becoming more affordable, and with epigenomic studies additional predisposing and causal factors will likely be identified in the future. It remains difficult though to ascertain the precise consequences of the methylation changes at individual DMRs. With the advent of CRISPR-based technologies to alter methylation at a locus of interest [130], also this question is open for research. 

Lastly, we now know that DNA methylation is not the only epigenetic modification that confers allelic biases in gene expression in the embryo. The recent mouse and human studies on PRC2-mediated patterns of H3K27me3 in oocytes show that this covalent histone modification confers allelic expression as well, in the early preimplantation embryo. In addition to its involvement in imprinted X-inactivation, and maintained imprinted expression of a few genes beyond implantation, through acquisition of allelic DNA methylation (Figure 1), the biological and clinical relevance of this transient type of imprinting remains unclear.

More and more insights are emerging through studies that did not initially focus on genomic imprinting. Between different systems of mono-allelic gene expression that evolved in mammals [11], striking mechanistic similarities have emerged that allow new candidates to be tested for their roles in imprinting. One example is the hinge domain protein SMCHD1, which is important not only for DNA methylation at sets of CpG islands on the inactive X chromosome in female cells, but is also required for gene methylation at the *Snrpn* imprinted domain on mouse chromosome 7 [53]. These are stimulating times for research on histone and DNA methylation imprints, on how these are maintained somatically, and how they confer mono-allelic expression and diverse biological roles. The rapidly expanding body of knowledge will undoubtedly yield a better understand of genomic imprinting and imprinting disorders during the years to come.

## Figures and Tables

**Table 1:** Proteins in the maintenance of DNA methylation imprints (adapted from [15]).

Protein	Description	Methylation Phenotype due to Loss of Expression, or Knock-Down, in Somatic Cells or Embryos	References
**DNA Methyltransferases (DNMTs) and Other DNA Methylation-Related Proteins:**			
DNMT1	maintenance DNA methyltransferase	Loss of imprinting control region (ICR) DNA methylation.	[32]
DNMT3A DNMT3B	*de novo* DNA methyltransferases	*Dnmt3a−/−* embryonic stem cells (ES) cells: ICR hypomethylation Double-Knock out (KO)embryos: partial loss of ICR methylation at *Rasgrf1* and *Peg3.*	[32,34]
UHRF1 (NP95)	binds hemi-methylated DNA post-replication and recruits DNMT1	Loss of ICR methylation.	[35]
MBD3	methyl CpG-binding domain protein-3	Loss of ICR methylation at *Igf2*-*H19* locus.	[36]
ZFP57	Krüppel associated box (KRAB) domain zinc finger protein	Reduced methylation at human ICRs (*PLAGL1, GRB10,* and *PEG3* loci).	[37,38]
ZFP445	KRAB domain zinc finger protein	Human embryonic stem cells (ESCs): loss of ICR methylation at *MEG3* and *IGF2-H19* loci. Zfp445−/− Zfp57−/− double KO embryos: loss of methylation at almost all ICRs.	[39]
KAISO (ZBTB33)	Zinc Finger and Broad-complex, Tramtrack and Bric- à brac (BTB) Domain Containing-33	Loss of ICR methylation at the human *IGF2-H19* imprinted locus.	[40]
**Histones and Other Chromatin-Related Proteins:**			
Histones H1	linker histones	Triple knockout (*H1c,d,e*): reduced ICR methylation at *Igf2-H19* and *Dlk1-Dio3.*	[41]
SETDB1	H3 lysine-9-specific histone methyl-transferase	*Loss of H3K9me3 at ICRs, loss of DNA methylation at Meg3*, *Nespas*, *Mest*, *Peg3.*	[42,43]
DPPA3 (PGC7, Stella)	methylated histone (H3K9me2) binding protein	Partial loss of DNA methylation at several ICRs.	[44,45]
KAP1 (TRIM28)	KRAB-associated protein 1	Partial loss of methylation at several ICRs (*Igf2*-*H19* and *Snrpn* loci).	[46]
ATRX	H3.3 histone chaperone	Loss of histone variant H3.3 and H3K9me3 at ICRs; no reported effects on DNA methylation.	[47]
**Other Proteins:**			
NAA10P (ARID1)	N-alfa-acetyltransferase 10	Loss of ICR/DMR methylation at *Trappc9-Peg13, Kcnq1-Kcnq1ot1, Mest, Snrpn, Grb10* and *H19*.	[48]
NLRP2	cytoplasmic caterpillar family protein	Loss of methylation at ICR of *KCNQ1* domain.	[49]
NLRP5	cytoplasmic caterpillar family protein	Decrease in methylation *PLAGL1, IGF2R, GRB10, MEST, KCNQ1OT1, MEG3* and *PEG3.*	[50]
MTA2	Metastasis tumor antigen-2	Partial losses of ICR methylation at *Igf2-H19* and *Peg3* domains.	[51]
RBBP1 and RBBP1L1	Retinoblastoma (Rb)-binding proteins	Combined knockout: loss of ICR methylation at the Prader-Willi syndrome (PWS) (*Snrpn)* imprinted gene domain.	[52]
SMCHD1	Hinge domain protein	ICR hypo-methylation at *Peg12* and *Snrpn.*	[53]
